# Supplementary material for: Expressed fusion gene landscape and its impact in multiple myeloma
Source: Nat Commun. 2017 Dec 1;8:1893. doi: 10.1038/s41467-017-00638-w (PMC5711960; doi:10.1038/s41467-017-00638-w)
Supplement: Supplementary file 1 — Supplementary Information [file 41467_2017_638_MOESM1_ESM.pdf]

File name: Supplementary Information
Description: Supplementary Figures and Supplementary Tables

File name: Supplementary Data 1
Description: Fusion list in MM cohort

File name: Supplementary Data 1
Description: Commands used for data analysis

File name: Peer Review File
Description:

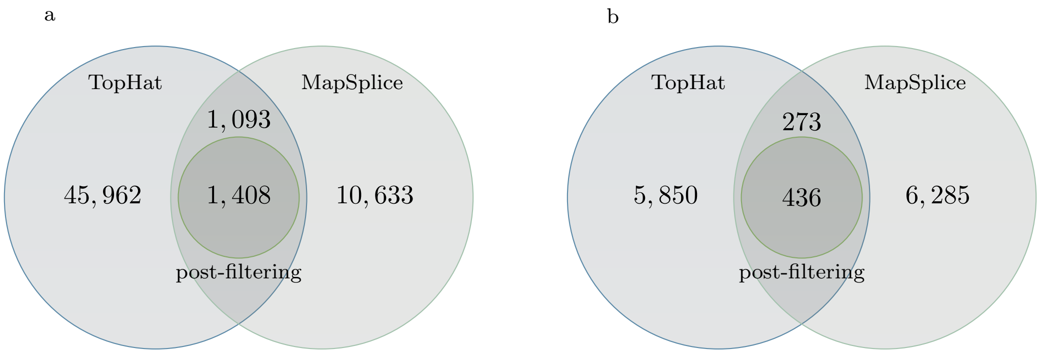


Supplementary Figure 1 – Number of fusions predicted by each algorithm, number found by both algorithms, and number of fusions retained post-filtering. In the IFM cohort (a) and in the cell-lines (b).

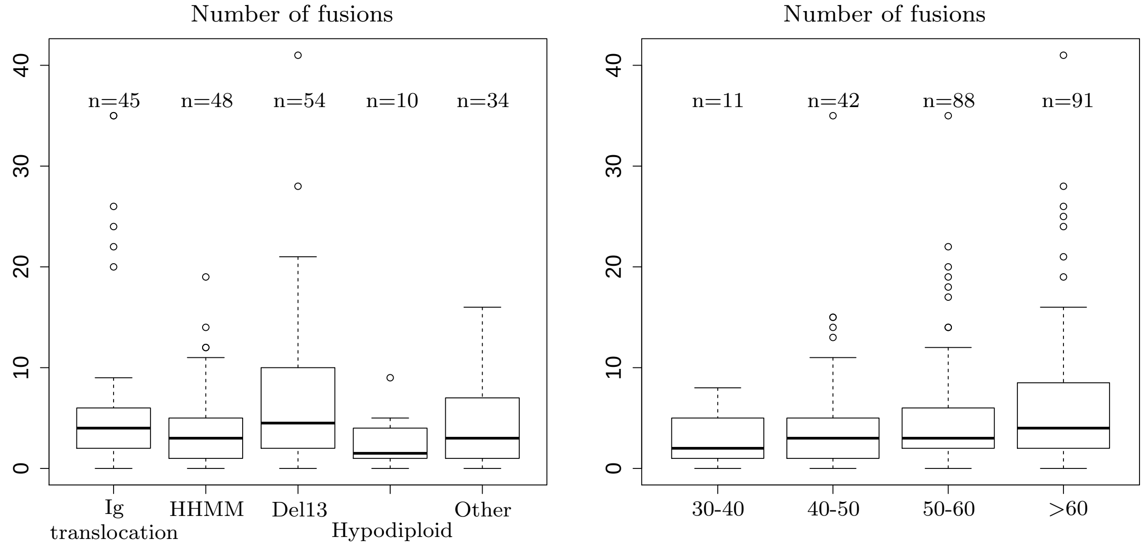


Supplementary Figure 2 – a : Boxplots of the number of fusions in each cytogenetic subgroup : known Ig-translocated (FISH experiments), high-hyperdiploid patients (over 53 chromosomes), patients with deletion 13, hypodiploid patients (less than 45 chromosomes) without deletion 13, and other patients. b : Number of fusions according to age.

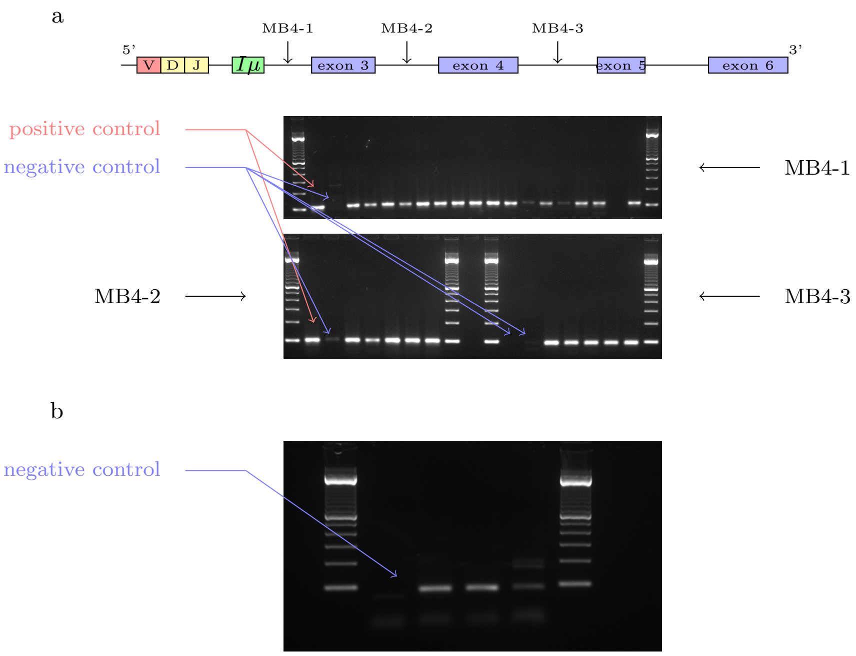


Supplementary Figure 3 – PCR validation of fusion genes. a : RT-PCR validation of IGH-MMSET fusions, with separation based on the breakpoint location : MB4-1 (MMSET intron n°2), MB4-2 (MMSET intron n°3) and MB4-3 (MMSET intron >4). b : subset of RT-PCR validation of IGH-B2M fusions.

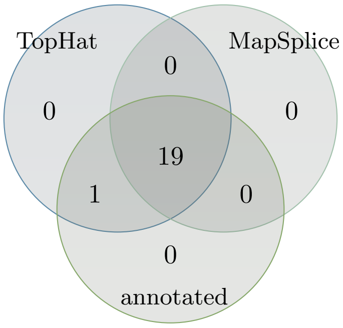


Supplementary Figure 4 – Number of IgH-MMSET fusions found by algorithms in the cell-lines known to exhibit a t(4;14) fusion. One sample was identified by TopHat only with evidence of the fusion occurring within an MMSET intron.

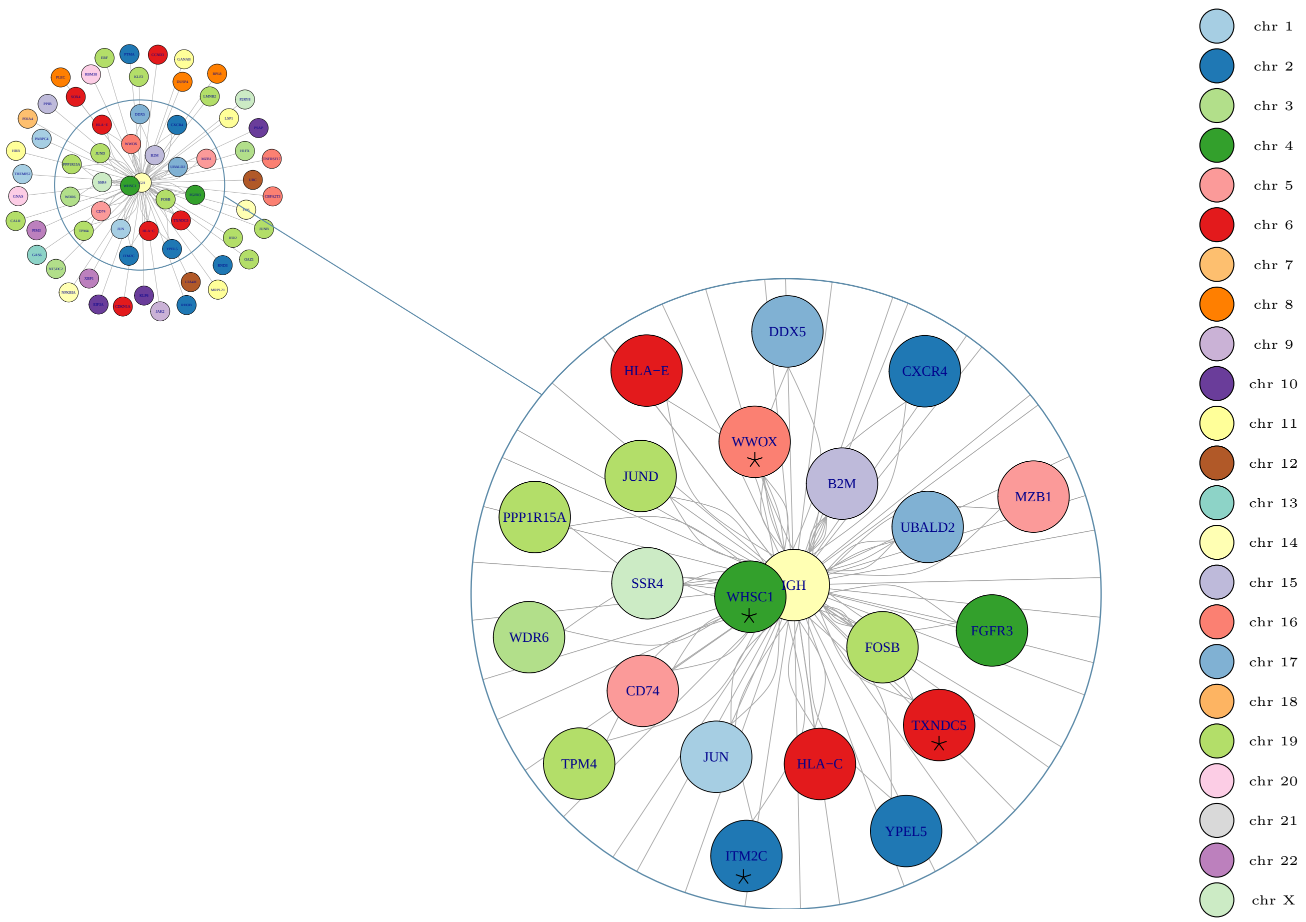


Supplementary Figure 5 – Most recurrent partner genes in IgH fusions. This figure is a zoom into main figure 3a. Stars denote fusions that were also found in cell-lines.

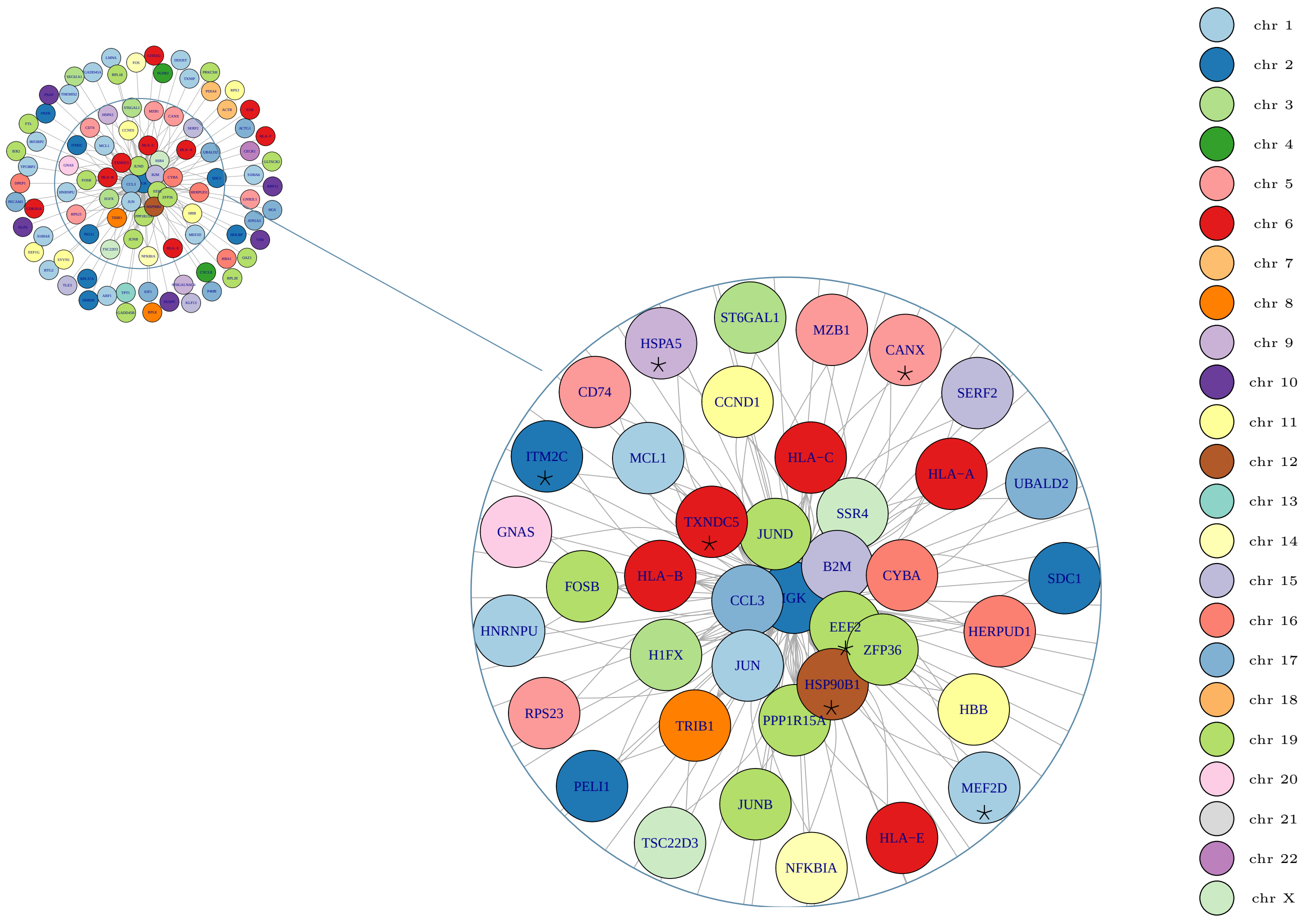


Supplementary Figure 6 – Most recurrent partner genes in IgK fusions. This figure is a zoom into main figure 3b. Stars denote fusions that were also found in cell-lines.

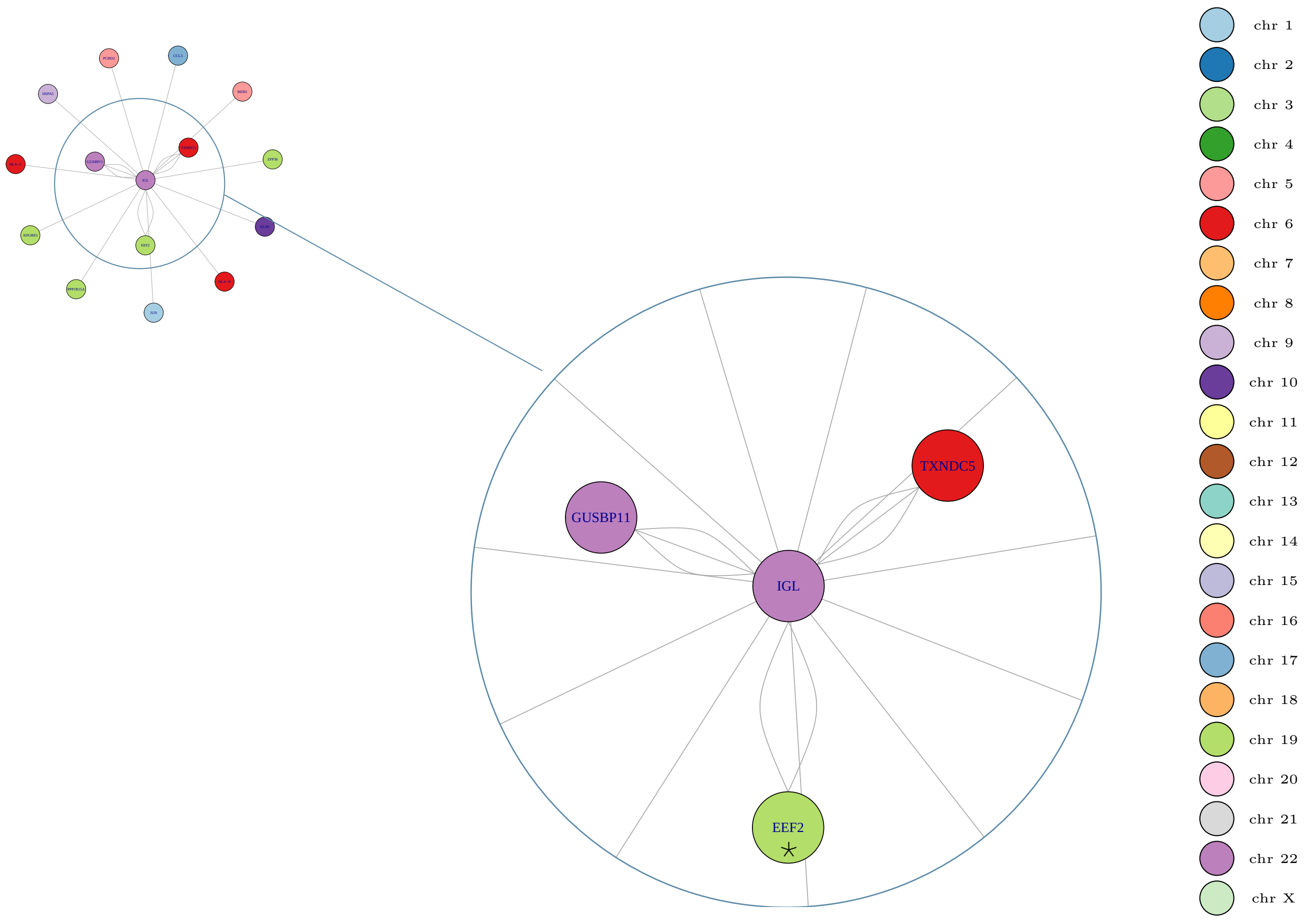


Supplementary Figure 7 – Most recurrent partner genes in IgL fusions. This figure is a zoom into main figure 3c. Stars denote fusions that were also found in cell-lines.

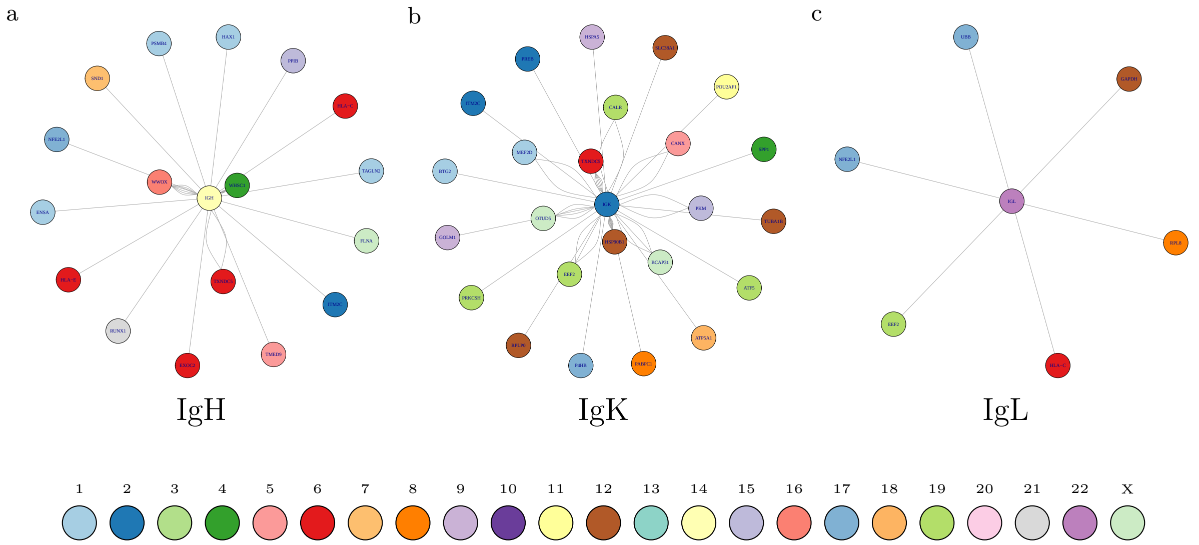


Supplementary Figure 8 – a : IgH fusions in cell-lines. The center edge is IgH, each other edge represents a partner gene, there is one node per fusion. Edges closest to the center are most recurrent. Edges are colored by chromosomes as indicated in d. b : IgK fusions in cell-lines. (Same as in a, with center edge IgK). c : IgL fusions in cell-lines. (Same as in a, with center edge IgL).

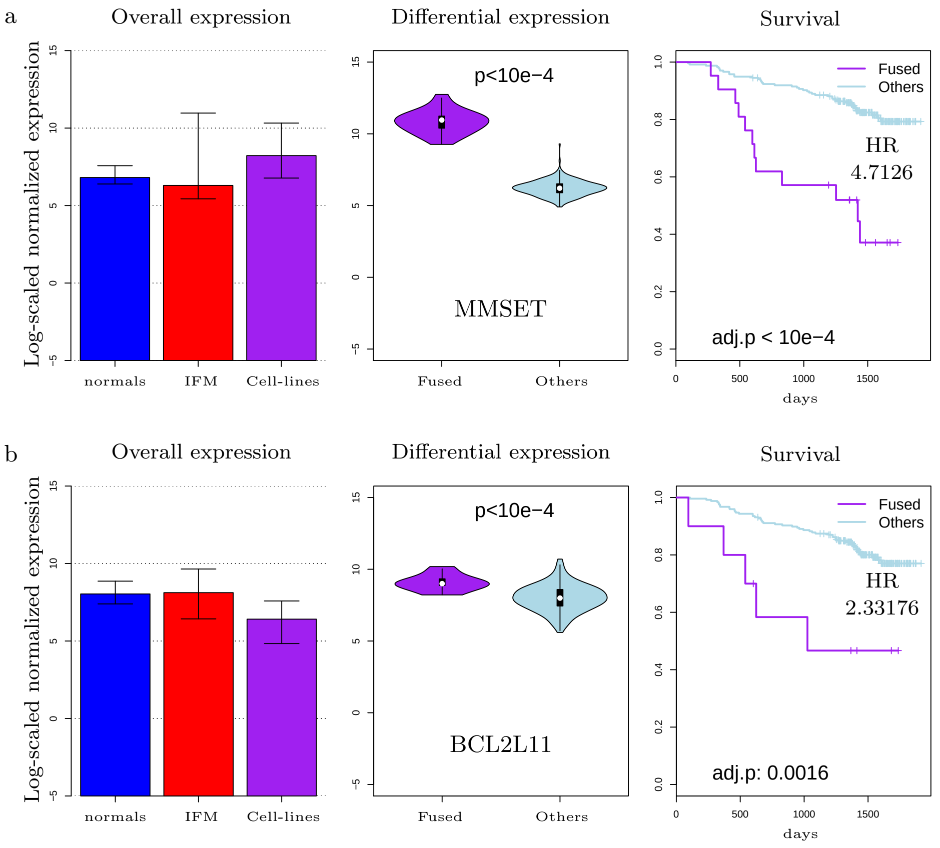


Supplementary Figure 9 – Two genes with clinical impact : MMSET (a) and BCL2L11 (b). The first sub-figure gives the expression of the gene in the normal samples, in the IFM cohort and in the cell-lines, error bars indicate standard deviation values. The second subfigure shows the distribution of gene expression within the IFM cohort, comparing patients with fusion (purple a) n=21, b) n=10), and patients without fusion (blue). The last subfigure gives the Kaplan-Meier estimate of the overall survival in the two previous subgroups, as well as the log-rank p-value adjusted for multiple testing on the 36 most frequently fused genes, and the hazard-ratio estimates from the Cox-proportional hazard model fitted with three high-risk known variables : ISS, presence of deletion 17p, and presence of t(4 ;14) translocation.

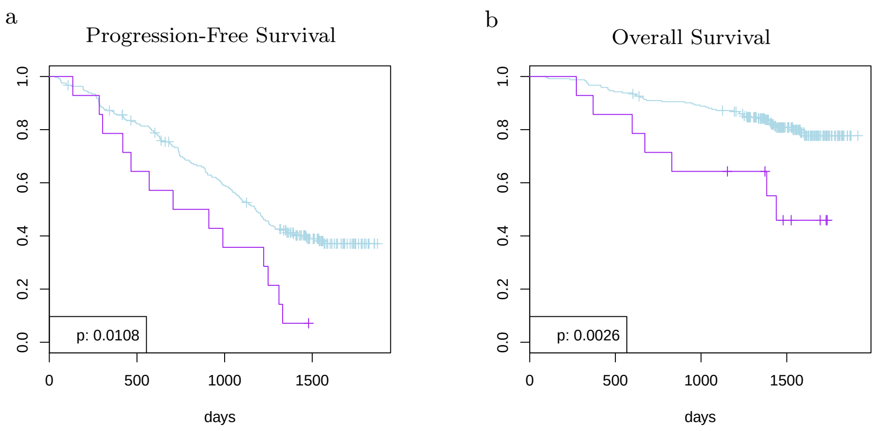


Supplementary Figure 10 – Clinical impact of the number of fusions : 16 patients with more than 16 fusions (a) Progression-free survival, (b) overall survival. P-values computed through log-rank tests.

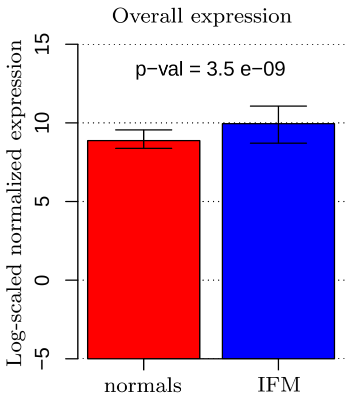


Supplementary Figure 11 – Expression of gene TXNDC5 in normal samples and in the IFM cohort, error bars indicate the 90% distribution interval. P-value computed using two-sided Student's t-test.

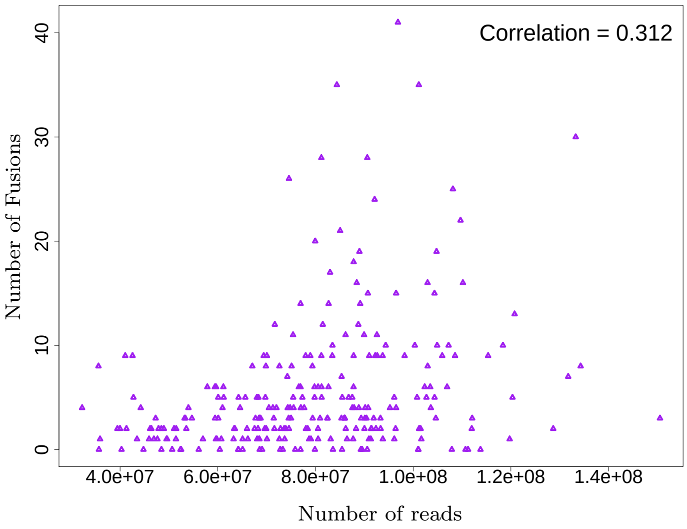


Supplementary Figure 12 – Correlation between sequencing depth (evaluated in terms of total amount of reads) and number of identified fusions, each triangle representing a patient sample.

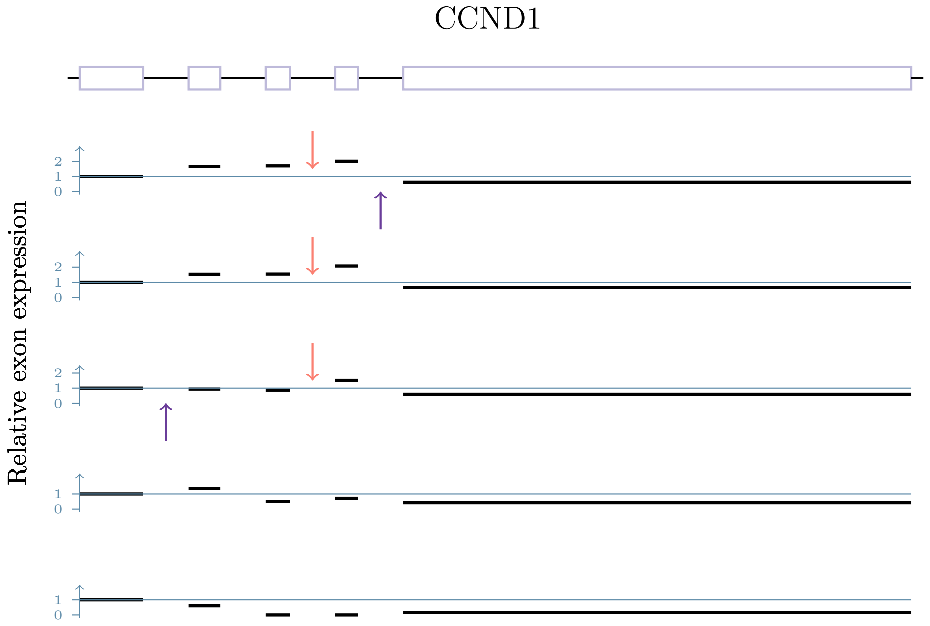


Supplementary Figure 13 – Coverage of CCND1 exons in 5 patients. Levels are computed based on read counts per exon, normalized by exon length and by first exon expression. Red arrows indicate “3prime” fusions, *i.e.* exons after the arrow are involved in a fusion. Purple arrows indicate “5prime” fusions, *i.e.* exons before the arrow are involved in a fusion. Patient 1 has both a 3prime and a 5 prime fusion, Patient 2 only has a 3prime fusion, and Patient 3 has a 5prime fusion occurring before ahead of the 5 prime fusion. Patient 4 and 5 do not harbor any fusion. Expression levels do not contradict the presence of fusions, but do not support those either, especially when compared to expressions from non-fused patients.

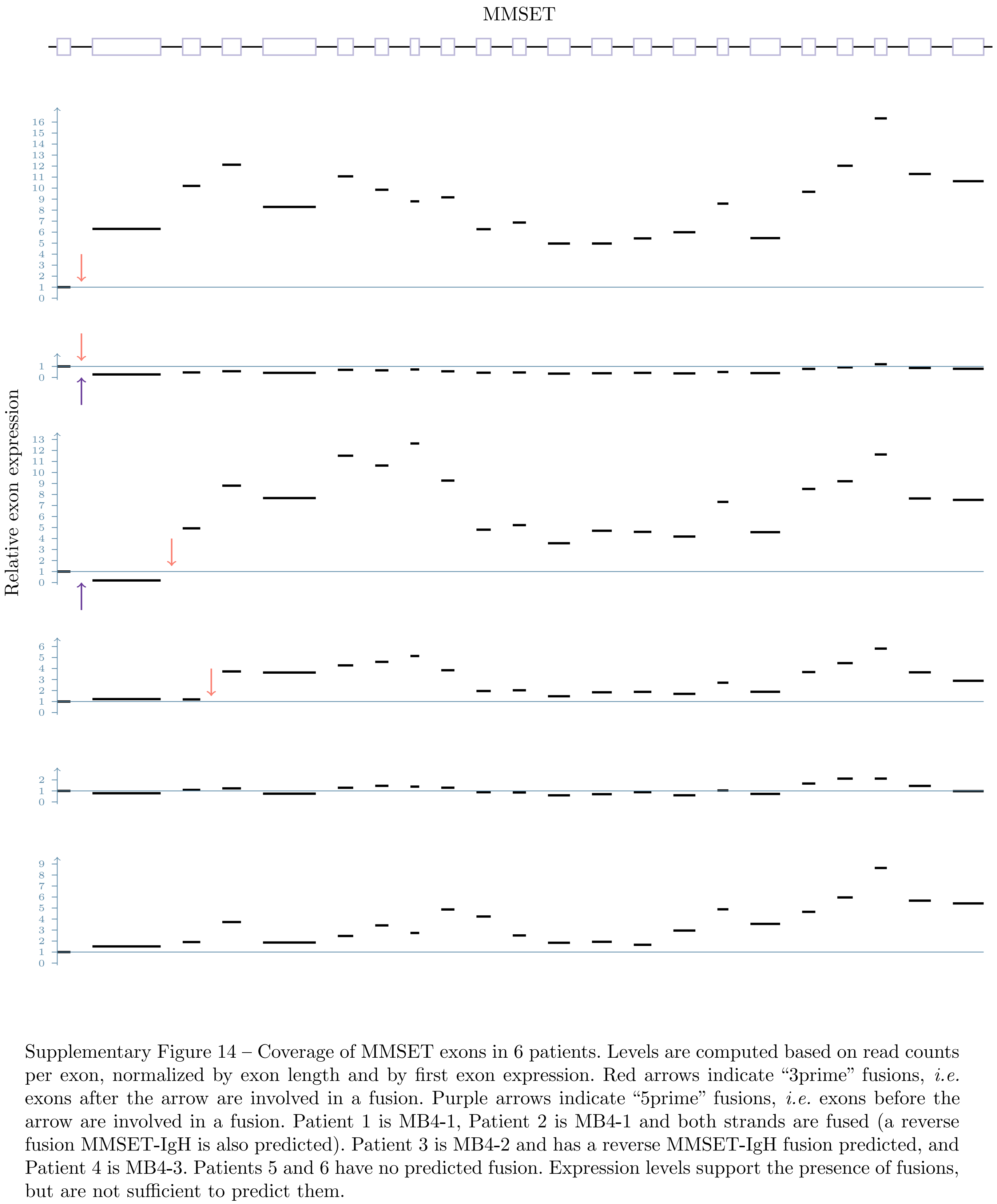


Supplementary Figure 14 – Coverage of MMSET exons in 6 patients. Levels are computed based on read counts per exon, normalized by exon length and by first exon expression. Red arrows indicate “3prime” fusions, *i.e.* exons after the arrow are involved in a fusion. Purple arrows indicate “5prime” fusions, *i.e.* exons before the arrow are involved in a fusion. Patient 1 is MB4-1, Patient 2 is MB4-1 and both strands are fused (a reverse fusion MMSET-IgH is also predicted). Patient 3 is MB4-2 and has a reverse MMSET-IgH fusion predicted, and Patient 4 is MB4-3. Patients 5 and 6 have no predicted fusion. Expression levels support the presence of fusions, but are not sufficient to predict them.

| | coef | exp(coef) | se(coef) | z | p |
|---|---|---|---|---|---|
| CSNK1G2_fusion | 1.08 | 2.95 | 0.31 | 3.47 | 0.00 |
| ISS | 0.21 | 1.24 | 0.13 | 1.67 | 0.10 |
| Del.17p | 0.50 | 1.65 | 0.27 | 1.86 | 0.06 |
| t.4.14 | 0.83 | 2.30 | 0.32 | 2.64 | 0.01 |

| | loglik | Chisq | Df | P(>\|Chi\|) |
|---|---|---|---|---|
| 1 | -541.62 | | | |
| 2 | -546.26 | 9.27 | 1 | 0.0023 |

Supplementary Table 1 – Output of fitted Cox-proportional hazard model and likelihood-ratio test for gene CSNK1G2 on progression-free survival

| | coef | exp(coef) | se(coef) | z | p |
|---|---|---|---|---|---|
| CCND1_fusion | 1.49 | 4.43 | 0.40 | 3.69 | 0.00 |
| ISS | 0.24 | 1.28 | 0.13 | 1.88 | 0.06 |
| Del.17p | 0.53 | 1.69 | 0.27 | 1.96 | 0.05 |
| t.4.14 | 0.80 | 2.21 | 0.31 | 2.53 | 0.01 |

| | loglik | Chisq | Df | P(>\|Chi\|) |
|---|---|---|---|---|
| 1 | -541.53 | | | |
| 2 | -546.26 | 9.46 | 1 | 0.0021 |

Supplementary Table 2 – Output of fitted Cox-proportional hazard model and likelihood-ratio test for gene CCND1 on progression-free survival

| | coef | exp(coef) | se(coef) | z | p |
|---|---|---|---|---|---|
| MMSET_fusion | 1.55 | 4.71 | 0.94 | 1.65 | 0.10 |
| ISS | 0.17 | 1.18 | 0.23 | 0.73 | 0.47 |
| Del.17p | 0.82 | 2.27 | 0.41 | 2.02 | 0.04 |
| t.4.14 | -0.48 | 0.62 | 0.98 | -0.49 | 0.63 |

| | loglik | Chisq | Df | P(>\|Chi\|) |
|---|---|---|---|---|
| 1 | -181.13 | | | |
| 2 | -182.17 | 2.09 | 1 | 0.1480 |

Supplementary Table 3 – Output of fitted Cox-proportional hazard model and likelihood-ratio test for gene MMSET on overall survival

| | coef | exp(coef) | se(coef) | z | p |
|---|---|---|---|---|---|
| BCL2L11_fusion | 0.83 | 2.29 | 0.58 | 1.44 | 0.15 |
| ISS | 0.18 | 1.20 | 0.23 | 0.79 | 0.43 |
| Del.17p | 0.79 | 2.20 | 0.42 | 1.90 | 0.06 |
| t.4.14 | 0.85 | 2.33 | 0.45 | 1.86 | 0.06 |

| | loglik | Chisq | Df | P(>\|Chi\|) |
|---|---|---|---|---|
| 1 | -181.30 | | | |
| 2 | -182.17 | 1.74 | 1 | 0.1869 |

Supplementary Table 4 – Output of fitted Cox-proportional hazard model and likelihood-ratio test for gene BCL2L11 on overall survival
